# Macrolide-Resistant *Mycoplasma pneumoniae* Infections among Children before and during COVID-19 Pandemic, Taiwan, 2017–2023

**DOI:** 10.3201/eid3008.231596

**Published:** 2024-08

**Authors:** Tsung-Hua Wu, Yu-Ping Fang, Fang-Ching Liu, Hui-Hsien Pan, Yu-Ying Yang, Chiah-Sing Song, Chun-Yi Lee

**Affiliations:** Show Chwan Memorial Hospital, Changhua, Taiwan (T.-H. Wu, Y.-Y. Yang);; College of Medicine, National Chung Hsing University, Taichung, Taiwan (T.-H. Wu, C.-Y. Lee);; Chang Bing Show Chwan Memorial Hospital, Changhua (Y.-P. Fang, C.-S. Song, C.-Y Lee);; Jen-Ai Hospital, Taichung (F.-C. Liu); Taichung Veterans General Hospital, Taichung (H.-H. Pan)

**Keywords:** pneumonia, antimicrobial resistance, COVID-19, Mycoplasma pneumoniae, bacteria, respiratory infections, severe acute respiratory syndrome coronavirus 2, SARS-CoV-2, SARS, coronavirus disease, zoonoses, viruses, coronavirus, macrolide resistance, molecular epidemiology, multilocus sequence typing, Taiwan

## Abstract

Before the COVID-19 pandemic, *Mycoplasma pneumoniae* infections emerged during spring to summer yearly in Taiwan, but infections were few during the pandemic. *M. pneumoniae* macrolide resistance soared to 85.7% in 2020 but declined to 0% during 2022–2023. Continued molecular surveillance is necessary to monitor trends in macrolide-resistant *M. pneumoniae*.

*Mycoplasma pneumoniae* is a major cause of respiratory tract infections, particularly in children and young adults. *M. pneumoniae* accounts for 10%–30% of community-acquired pneumonia (*1*). Macrolides are the primary treatment, but since 2000, macrolide-resistant *M. pneumoniae* (MRMP) strains have increased substantially, especially in Asia (*2*,*3*). However, a global study indicated a decline in *M. pneumoniae* detections during 2017–2021, which researchers attributed to the impact of nonpharmaceutical COVID-19 measures on *M. pneumoniae* transmission (*4*). 

In Taiwan, MRMP prevalence rose from 12.3%–24% before 2017 to 54%–88% during 2017–2020 (*5*–*7*). Multilocus sequence typing (MLST) is a valuable tool for epidemiologic surveillance, offering high discriminatory ability to identify the shift of circulating strain types (*8*–*10*). We used MLST to analyze the genetic diversity and macrolide resistance prevalence of *M. pneumoniae* in hospitalized children in Taiwan during 2017–2023. This study was approved by the institutional review board of Show Chwan Memorial Hospital (approval no 1051007 and 1091104).

## The Study

Our study spanned 2 phases: phase 1 was March 2017–June 2019, and phase 2 was March 2020–December 2023. After obtaining the necessary consent, we enrolled children <18 years of age at 4 central hospitals in Taiwan: Show Chwan Memorial Hospital (739 beds), Chang Bing Show Chwan Memorial Hospital (946 beds), Jen-Ai Hospital (644 beds), and Chung Shan Medical University Hospital (1,023 beds). We enrolled children with acute respiratory tract infections who tested *M. pneumoniae* IgM–positive via Biocardä (AniBiotech, https://anibiotech.fi). We collected nasopharyngeal or oropharyngeal swab specimens, stored them, and cultured for *M. pneumoniae*. We performed MLST typing on positive cultures confirmed by PCR as true *M. pneumoniae* infections. 

We cultured specimens in SP-4 medium and incubated at 37°C in 5% CO_2_ for 14 days (*11*). We used the QIAamp DNA Blood Mini Kit (QIAGEN, https://www.qiagen.com) to extract DNA and confirmed *M. pneumoniae* by real-time PCR targeting the repMp1 gene (*12*). We further analyzed *M. pneumoniae* isolates, including single-base mutations in the 23S rRNA gene and MLST on the basis of 8 housekeeping genes (*8*). We used goeBURST software (https://phyloviz.readthedocs.io/en/latest/data_analysis.html) to explore relationships among sequence types (STs).

**Figure 1:**
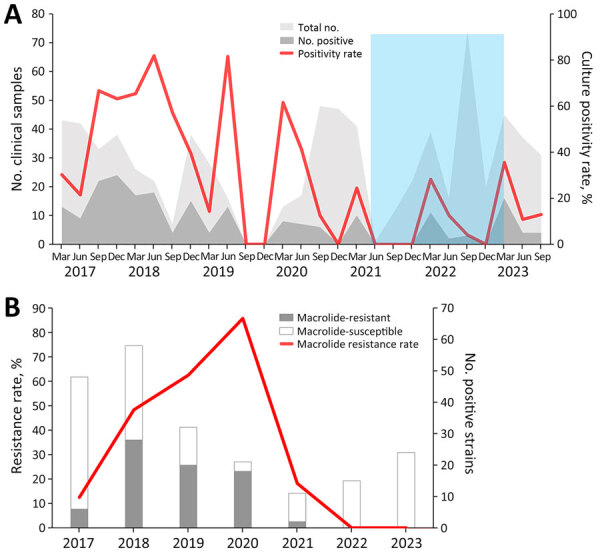
Dynamic distribution of macrolide-resistant *Mycoplasma pneumoniae* infections among children before and during the COVID-19 pandemic, Taiwan, 2017–2023. A) *M. pneumoniae* infections were detected throughout the year, primarily from March to August. An *M. pneumoniae* outbreak occurred during 2017–2018. The *M. pneumoniae* detection rate substantially declined during the COVID-19 pandemic, 2021–2022. Light gray background represents 770 IgM-positive participants; dark gray background represents 209 cases confirmed by culture and PCR. Blue shading indicates timeframe of nonpharmaceutical interventions during the COVID-19 pandemic. B) Among 211 isolates, macrolide resistance was observed in 74 (35.1%) isolates. The resistance rate was 12.5% in 2017, increased to 48.3% in 2018 and to 62.5% in 2019, and reached 85.7% in 2020. Subsequently, the rate decreased to 18.2% in 2021 and dropped to 0% in 2022 and 2023.

Among 770 nasopharyngeal or oropharyngeal samples collected during the study period, 209 (27.1%) were confirmed *M. pneumoniae* cases. By comparing the annual and seasonal distribution of *M. pneumoniae* respiratory tract infections, we noted significant yearly differences (p<0.001) and an outbreak that occurred during 2017–2018 (Table 1). Seasonal prevalence also varied significantly (p<0.001); average positivity rates were 30% in spring, 41.6% in summer, 18.1% in autumn, and 22.1% in winter. *M. pneumoniae* was detected year-round, but primarily during March–August (Figure 1, panel A). During the COVID-19 pandemic, detection rates notably declined. 

**Table 1:** Annual and seasonal distribution of macrolide-resistant *Mycoplasma pneumoniae* infections among children before and during the COVID-19 pandemic, Taiwan, 2017–2023*

Year	No. positive cases (%)					p value†
	Total no. cases	Spring, Mar–May	Summer, Jun–Aug	Autumn, Sep–Nov	Winter, Dec–Feb	
2017	67 (43.23)	13 (30.23)	9 (21.43)	21 (65.63)	24 (63.16)	<0.001
2018	54 (58.06)	17 (65.38)	18 (81.82)	4 (57.14)	15 (39.47)	0.010
2019	17 (38.64)	4 (14.29)	13 (81.25)	ND	ND	NA
2020	21 (16.80)	8 (61.54)	7 (41.18)	6 (12.50)	0	<0.001
2021	10 (13.33)	10 (24.39)	0	0	0	0.016
2022	16 (11.59)	11 (28.21)	2 (12.50)	3 (4.05)	0	0.002
2023	24 (17.14)	5 (13.51)	13 (37.14)	3 (9.38)	3 (8.33)	0.008
Total	209 (27.14)	68 (29.96)	62 (41.61)	37 (18.14)	42 (22.11)	<0.001
p value‡	<0.001					

*The study period ended December 2023. NA, not applicable; ND, no data. †p value by Fisher exact test to examine the relationship between season and infection. ‡p value by Χ^2^ test to examine the relationship between year and infection.

Among the 209 *M. pneumoniae–*positive isolates, 74 (35.4%) were macrolide-resistant. Annual resistance rates ranged from 12.5% in 2017 to 85.7% in 2020, then dropped to 18.2% in 2021 and 0% in 2022 and 2023 (Figure 1, panel B). Among macrolide-resistant isolates, 72 (97.3%) had the A2063G mutation, and 2 (2.7%) had the A2063T mutation.

**Figure 2:**
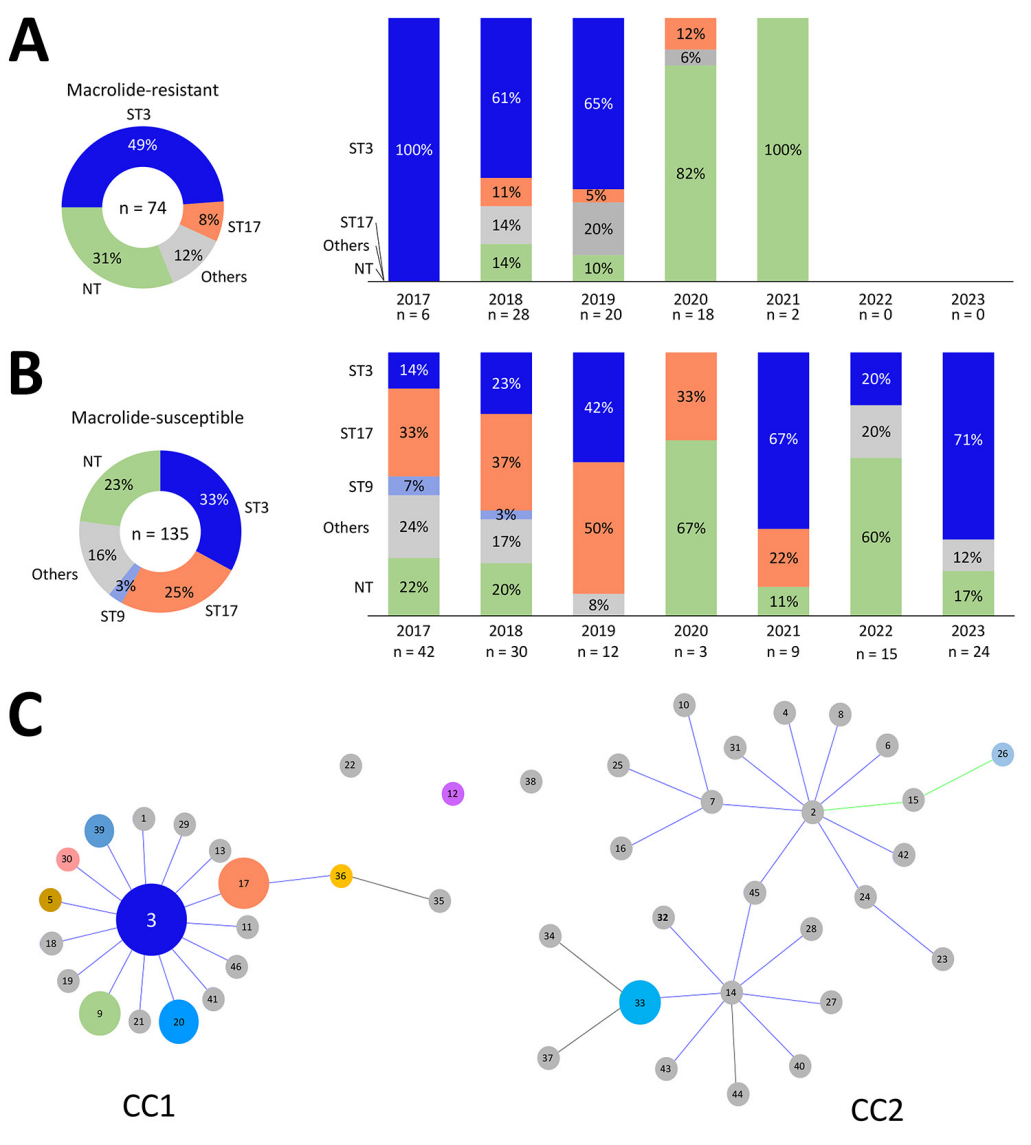
Relationships between year of isolation, ST, and genotype distribution in a study of macrolide-resistant *Mycoplasma pneumoniae* infections among children before and during the COVID-19 pandemic, Taiwan, 2017–2023. A) Macrolide-resistant *M. pneumoniae* isolates (n = 74) and 5 identified STs: ST3, ST17, ST26, ST33, and ST36. Macrolide resistance substantially decreased after 2021. ST3 was the predominant strain in macrolide-resistant isolates, especially during 2017–2019. B) Macrolide-susceptible *M. pneumoniae* isolates (n = 135) and 10 identified STs: ST3, ST17, ST9, ST5, ST12, ST20, ST30, ST33, ST39, and ST46. ST17 was the predominant strain during 2017–2019. ST3 was the most common strain and was distributed across all years. C) The relationship between *M. pneumoniae* CC and ST depicted by goeBURST (https://phyloviz.readthedocs.io/en/latest/data_analysis.html). The data, comprising 144 strains from Taiwan (2017–2023) and previously reported STs (shown in gray) from PubMLST (https://pubmlst.org), demonstrate the genetic relationships within the dataset analyzed by the goeBURST algorithm. The size of each circle is proportional to the number of isolates for each ST, and most STs belonged to CC1, including the leading 2 STs, ST3, and ST17. Green sections indicate *M. pneumoniae* strains could not be successfully identified using multilocus sequence typing. CC, clonal complex; NT, nontypable; ST, sequence type.

We submitted all 209 confirmed *M. pneumoniae* strains for molecular analysis, and 155 were identified by MLST, revealing 12 different STs (Appendix Table). ST3 was the most prevalent at 38.2%, followed by ST17 at 19.1%. However, 54 (25.8%) strains could not be successfully genotyped due to the failure of amplification and sequencing of the *pgm* locus. Of the 74 macrolide-resistant isolates, the leading STs were ST3 (49%) and ST17 (8%), but ST3 was the most common in MRMP during 2017–2019 (Figure 2, panel A). Among 135 macrolide-susceptible isolates, ST33 (33%), ST17 (25%), and ST9 (3%) were predominant (Figure 2, panel B). ST17 was more common in macrolide-susceptible than in macrolide-resistant isolates (p = 0.003), and ST3 was more prevalent in macrolide-resistant isolates (p = 0.002). However, we found no correlation between the dynamic proportion of macrolide resistance and ST3, although a notable percentage of macrolide-resistant strains within ST3 were detected during 2017–2019 (Table 2).

**Table 2:** Distribution of macrolide susceptibility of sequence type 3 and 17 isolates in a study of macrolide-resistant *Mycoplasma pneumoniae* infections among children before and during the COVID-19 pandemic, Taiwan, 2017–2023

Macrolide susceptibility	Year, no. (%) isolates						
	2017	2018	2019	2020	2021	2022	2023
Sequence type 3							
Resistant	6 (50)	17 (70.8)	13 (72.2)	0	0	0	0
Susceptible	6 (50)	7 (29.2)	5 (27.8)	0	6 (100)	3 (100)	17 (100)
Total no. isolates	12	24	18	0	6	3	17
Sequence type 17							
Resistant	0	3 (21.4)	1 (14.3)	2 (66.7)	0	0	0
Susceptible	14 (100)	11 (78.6)	6 (85.7)	1 (33.3)	2 (100)	0	0
Total no. isolates	14	14	7	3	2	0	0

We identified 2 clonal complexes (CCs) in the goeBURST analysis (Figure 2, panel C), which included data from 140 strains. Of those 2 CC clusters, most (96%) STs belonged to CC1, which included the most frequently detected STs, ST3 and ST17. CC2 comprised 5 (4%) strains and included 2 STs, ST33 and ST26.

## Conclusions

During 2017–2023, we observed changes in *M. pneumoniae* infection rates, alterations in STs, and shifts in antimicrobial resistance. Nonpharmaceutical interventions during the pandemic mitigated the transmission of respiratory pathogens besides SARS-CoV-2 (*13*). In our study, we noted a substantial decrease in *M. pneumoniae* detection during 2021, and no positive cases were recorded during June 2021–February 2022, coinciding with the height of the pandemic period in Taiwan, a finding that aligns with those reported in a global survey (*4*).

MRMP infections have increased greatly worldwide, particularly in the Western Pacific region (*14*). In Taiwan, prior studies noted a substantial rise in macrolide resistance rates from 12%–24% during 2011–2016 to 54%–88% during 2017–2020 (*5*–*7*). However, in our study, MRMP prevalence decreased rapidly to 0%–18.2% during 2021–2023. During the 2011–2012 outbreak in Japan, the MRMP detection rate soared to 90% (*9*). After that outbreak, the number of MRMP strains decreased, reaching 14.3% in 2018, whereas China and South Korea continued to report high resistance rates from 2014 to 2018 (*10*,*15*).

ST3 within CC1 is a globally successful clone and was prevalent in Japan, China, South Korea, Cuba, Germany, and Taiwan at rates from 30% to 70% during 2002–2022 (Appendix Figure). A previous study in Taiwan showed high macrolide resistance rates for ST3 (93.5%) and ST17 (82.1%) (*7*). In contrast, ST17 was more common among the macrolide-susceptible cases in our study and had a resistance rate of only 15%. Recent data indicate shifts in *M. pneumoniae* sequence types: in Japan, ST3 and ST14 were largely replaced by ST7 and ST33 in 2018–2019, reducing macrolide resistance by 11.3% (*9*). However, South Korea did not have a notable decrease in its macrolide resistance rate (78.5%) during 2019–2020, and ST3 remained dominant (*10*). Our findings show no substantial shifts in ST distribution in Taiwan but a notable change in the ratio of macrolide-resistant to macrolide-susceptible within ST3, possibly reducing the overall resistance rate (Table 2).

The first limitation of our study is that an 8-month gap in data collection occurred during July 2019–February 2020, which might have caused a slight underestimation of cases. Second, we only enrolled patients who tested positive for *M. pneumoniae* IgM, which might underestimate the actual number of *M. pneumoniae* infections. We did not investigate the clinical manifestations in patients who tested IgM-negative. Third, although the trend toward resistant strains was evident, the absolute number of resistant strains was small. Fourth, some strains failed at the *pgm* locus, and on the basis of other known loci, those strains are most likely to be either ST3 or ST17. That issue might have been to the result of low bacterial loads and fragile DNA that hindered the successful amplification of the 1,072-bp *pgm* locus via nested PCR. Recent advances have made whole-genome sequencing a promising tool for future *M. pneumoniae* research, especially in identifying genotypes linked to macrolide resistance or virulence.

In conclusion, *M. pneumoniae* respiratory tract infections in Taiwan exhibit seasonality, and prevalence decreased after the COVID-19 pandemic. Simultaneously, MRMP incidence experienced a sharp decline after 2021. Although ST3 remains the most prevalent *M. pneumoniae* strain and is associated with macrolide resistance, global data still lack evidence of correlation between STs and macrolide resistance. Thus, continued molecular surveillance is necessary to monitor those trends.

Appendix Additional information on macrolide-resistant *Mycoplasma pneumoniae* infections among children before and during the COVID-19 pandemic, Taiwan, 2017–2023.
