# Supplementary material for: Macrolide-Resistant Mycoplasma pneumoniae Infections among Children before and during COVID-19 Pandemic, Taiwan, 2017–2023
Source: Emerg Infect Dis. 2024 Aug;30(8):1692–6. doi: 10.3201/eid3008.231596 (PMC11286047; doi:10.3201/eid3008.231596)
Supplement: Appendix — Additional information on macrolide-resistant Mycoplasma pneumoniae infections among children before and during the COVID-19 pandemic, Taiwan, 2017–2023. [file 23-1596-Techapp-s1.pdf]

Article DOI: https://doi.org/10.3201/eid3008.231596
EID cannot ensure accessibility for supplementary materials supplied by authors. Readers who have difficulty accessing supplementary content should contact the authors for assistance.

Macrolide-Resistant *Mycoplasma pneumoniae* Infections among Children before and during COVID-19 Pandemic, Taiwan, 2017–2023

Appendix

Appendix Table. Relationship between year of isolation and sequence type (ST) among *M. pneumoniae* isolates, 2017–2023

Year	ST3	ST17	ST9	ST20	ST33	Others*	Nontypable	Total
2017	12	14	3	0	2	8	9	48
2018	24	14	1	0	2	7	10	58
2019	18	7	0	0	0	5	2	32
2020	0	3	0	0	0	1	17	21
2021	6	2	0	0	0	0	3	11
2022	3	0	0	3	0	0	9	15
2023	17	0	0	1	0	2	4	24
Total	80	40	4	4	4	23	54	209

*Other STs include ST5, ST12, ST26, ST30, ST36, ST39, and ST46.

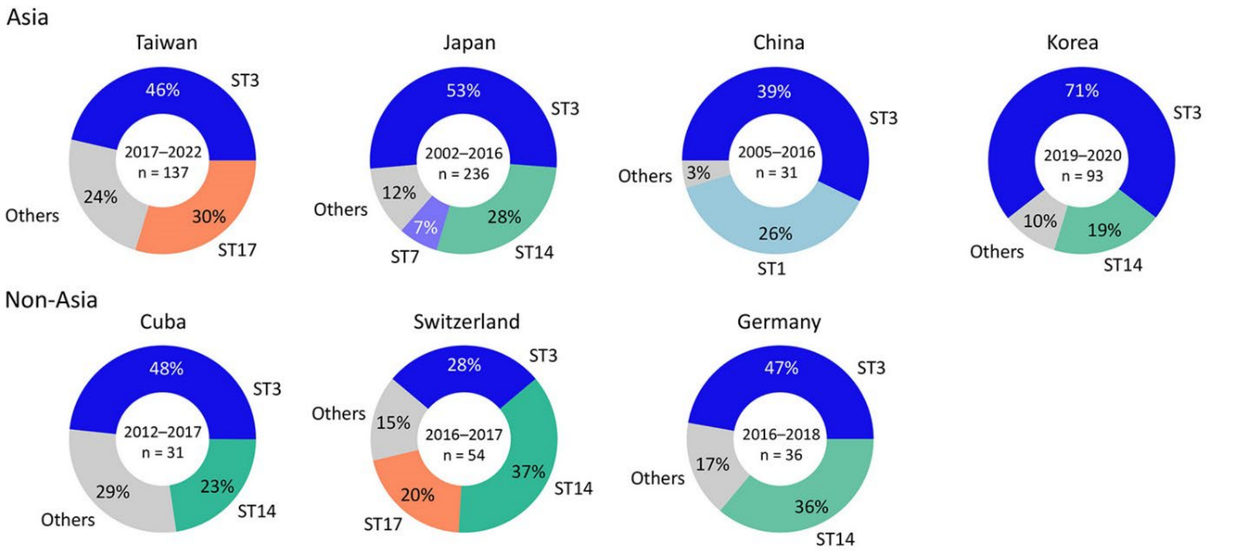

Appendix Figure. The distribution of *M. pneumoniae* strains from Japan (*1*), China (*2*), South Korea (*3*), Cuba (*4*), Switzerland (*5*), Germany (*5*), and Taiwan (*2*), 2002–2022. ST3 was the predominant strain in these countries, while other strains vary from one country to another. ST, sequence type.

References

1. Ando M, Morozumi M, Adachi Y, Ubukata K, Iwata S. Multilocus sequence typing of *Mycoplasma pneumoniae*, Japan, 2002–2016. Emerg Infect Dis. 2018;24:1895–901. PubMed https://doi.org/10.3201/eid2410.171194

2. Hsieh YC, Li SW, Chen YY, Kuo CC, Chen YC, Chang IY, et al. Global genome diversity and recombination in *Mycoplasma pneumoniae.* Emerg Infect Dis. 2022;28:111–7. PubMed https://doi.org/10.3201/eid2801.210497

3. Lee JK, Choi YY, Sohn YJ, Kim KM, Kim YK, Han MS, et al. Persistent high macrolide resistance rate and increase of macrolide-resistant ST14 strains among *Mycoplasma pneumoniae* in South Korea, 2019–2020. J Microbiol Immunol Infect. 2022;55:910–6. PubMed https://doi.org/10.1016/j.jmii.2021.07.011

4. Dumke R, Rodriguez N. Use of different approaches for the culture-independent typing of *Mycoplasma pneumoniae* from two geographically distinct regions. J Microbiol Methods. 2021;186:106239. PubMed https://doi.org/10.1016/j.mimet.2021.106239

5. Meyer Sauteur PM, Pánisová E, Seiler M, Theiler M, Berger C, Dumke R. *Mycoplasma pneumoniae* genotypes and clinical outcome in children. J Clin Microbiol. 2021;59:e0074821. PubMed https://doi.org/10.1128/JCM.00748-21
